# Switching lasers: assessing the learning curves of surgeons with different levels of surgical experience when switching from HoLEP to pulsed Thulium YAG lasers for ThuLEP

**DOI:** 10.3389/fsurg.2026.1799916

**Published:** 2026-04-13

**Authors:** Leo F. Stadelmeier, Laurenz Berger, Philip Nicola, Philipp Weinhold, Julian Marcon, Michael Atzler, Yannic T. Volz, Nikolaos Pyrgidis, Iason Papadopoulos, Martin Hennenberg, Christian G. Stief, Patrick M. Keller, Alexander Tamalunas

**Affiliations:** Department of Urology, University Hospital of Munich, Munich, Germany

**Keywords:** BPE, BPH, HoLEP, laser, LUTS, surgery, ThuLEP

## Abstract

**Objectives:**

This study aims to assess the learning curves associated with pulsed Thulium laser enucleation of the prostate (ThuLEP) among three surgeons with varying levels of experience in Holmium laser enucleation of the prostate (HoLEP) as a treatment for lower urinary tract symptoms (LUTS) secondary to benign prostatic enlargement, with pulsed ThuLEP being one of the newest systems for the surgical treatment of male LUTS.

**Methods:**

We conducted a prospective analysis of the first 100 consecutive ThuLEP procedures performed by three surgeons with varying levels of HoLEP experience: one highly experienced (>1,000 prior HoLEP surgeries), one moderately experienced (>200 prior HoLEP surgeries), and one novice surgeon (with no prior HoLEP surgeries) undergoing a structured training program. The evaluation focused on perioperative characteristics, functional results, and safety outcomes.

**Results:**

While postoperative functional outcomes were comparable across all groups, experienced surgeons demonstrated a steeper learning curve. The highly experienced surgeon achieved proficiency approximately twice as quickly as the moderately experienced one. Surgeons with prior HoLEP experience reached a performance plateau in enucleation efficiency (g/min) and enucleation time (min) roughly twice as quickly, while requiring only about half the laser energy (kJ). Training a HoLEP-inexperienced surgeon in ThuLEP proved both feasible and safe when conducted within a structured training program.

**Conclusions:**

Pulsed ThuLEP shows a learning curve comparable to HoLEP for inexperienced surgeons when performed following a structured training program. Switching lasers is safe and feasible for surgeons already experienced in HoLEP.

## Introduction

Benign prostatic enlargement (BPE), resulting from benign prostatic hyperplasia (BPH), a non-malignant growth of prostatic tissue, is a common concomitant of aging (1).

Although pharmacological treatment options are available, contraindications, limited efficacy, and high discontinuation rates of up to 90% highlight the need for surgical options (2, 3).

Today, a broad range of surgical and interventional methods for the treatment of benign prostatic obstruction (BPO) due to BPE, including transurethral electric resection of the prostate (TUR-P), vaporization, or Holmium laser enucleation of the prostate (HoLEP), have been developed and incorporated into international guidelines (4–6). Since its introduction in the 1990s, laser enucleation of the prostate has established itself as an effective and safe method for the treatment of BPE-related LUTS. Multiple studies have demonstrated the efficacy and safety of various enucleation techniques compared with TUR-P, the former gold standard, regardless of patient age and prostate size (7–10). A related technique, using a different laser agent, is Thulium laser enucleation of the prostate (ThuLEP), which has been introduced in recent years (11). Multiple studies have shown its effectiveness including a comparison with other treatment options for benign prostatic obstruction, such as transurethral resection of the prostate or HoLEP (12, 13). This led to ThuLEP and HoLEP being recognized as well-established surgical methods, with HoLEP being favored over monopolar transurethral resection of the prostate (mTURP) in some cases due to its lower morbidity (4).

While ThuLEP is not only a viable option for the treatment of benign prostatic enlargement in terms of therapeutic outcomes, its practicability has also been well tested in studies focusing on learning curves while training (14–16).

However, while the advantages are self-evident, steep learning curves, combined with the high costs associated with the acquisition and maintenance of these systems, have slowed the widespread adoption of the various laser systems (16).

Pulsed solid-state Thulium:Yttrium Aluminum Garnet (YAG) lasers represent a novel alternative and, due to their high peak energy rates, show considerable promise for various endourological applications and have been among the most anticipated novelties in recent years (17–19).

A systematic review by Uleri et al. demonstrated comparable safety and functional outcomes across different laser platforms, highlighting that differences between technologies are often more related to technical characteristics and surgeon familiarity than to clinically meaningful differences in outcomes, hinting at the opportunity for experienced surgeons to quickly adapt to novel systems (20). While several studies have focused on learning curves for both Holmium and Thulium laser systems, and only one study has explored the learning curve when switching from Thulium to Holmium lasers, there is currently no study examining how surgeons with different levels of experience in Holmium laser techniques might be able to switch to a pulsed Thulium-based system (14–16, 21).

The main objective of this study was to assess the learning curve of surgeons with different levels of prior experience in HoLEP surgery after switching to a pulsed Thulium-based laser system. To achieve this, we prospectively analyzed efficacy, efficiency, and safety outcomes across three surgeons.

## Materials

### Study design and surgical procedures

For this study, the first 100 consecutive cases of pulsed ThuLEP performed by three surgeons were prospectively collected to analyze learning curves and assess individual performance. Surgeon 1 was a highly experienced HoLEP surgeon, having performed more than 1,000 HoLEP cases. Surgeon two was an experienced HoLEP surgeon, having performed more than 200 cases. Surgeon 3 had an extensive experience in endourological procedures and TUR-P but no prior HoLEP experience and underwent a structured training program as described by Westhofen et al. (16). The curriculum consists of a stepwise training program comprising approximately 50 supervised procedures, divided into three progressive training phases. Initially, the trainee performs 10 cases focusing on middle-lobe preparation, followed by 20 cases including lateral lobe enucleation, and subsequently 20 additional procedures emphasizing ventro-apical dissection of the adenoma. During this period, a HoLEP-experienced mentor completes the procedure after each respective training step is performed (16). Upon completion of these 50 supervised interventions and fulfillment of the procedural objectives, the trainee progresses to independent performance of the technique.

We included the first 100 consecutive patients treated by each surgeon who underwent ThuLEP for LUTS due to BPO at our institution between July 2023 and March 2024. Indications for ThuLEP were based on the current EAU guidelines for the management of non-neurogenic male LUTS (6). In line with previously published data, the three-lobe enucleation technique was applied consistently by all surgeons (7, 22).

The laser system used for all interventions was a pulsed solid-state Thulium:YAG laser (Thulio®, Dornier MedTech Systems GmbH, Weßling, Germany). Laser settings for tissue enucleation were 75 W, 1.5 J, and 50 Hz. Tissue morcellation was performed using the Piranha morcellator system (Richard Wolf GmbH, Knittlingen, Germany). All surgeons used the aforementioned equipment for every procedure.

After cystoscopy at the beginning of each procedure, a 26-Fr continuous-flow resectoscope was inserted. As part of the three-lobe technique, a laser bladder neck incision is made at the 5 o’clock position to define the capsular plane, followed by enucleation of the median lobe and then the lateral lobes separately. Morcellation, resection of remaining tissue, and coagulation are subsequently performed (22).

Our standard protocol includes the insertion of a 24-Fr three-way Foley catheter, followed by continuous irrigation of the bladder with saline solution for 12 h (7).

To ensure comparability, the laser system, laser settings, surgical technique, perioperative management, and postoperative irrigation protocol were kept unchanged for all cases included in the analysis.

### Parameters

Preoperative functional parameters were compared to those measured at 4 weeks after surgery. Prior to surgery, the International Prostate Symptom Score (IPSS), Quality of Life (QoL) score, peak urinary flow rate (Qmax, mL/s), and postvoid residual volume (PVR, mL) were examined and compared.

To assess perioperative performance, we evaluated preoperative prostate volume (PV, mL), determined by transrectal ultrasound (TRUS), total surgical time (min), resected prostate weight (g), tissue retrieval percentage (%), laser energy required (kJ), enucleation efficiency (g/kJ), enucleation efficacy (g/min), and hemoglobin (Hb) loss (g/dL).

Changes in functional parameters were assessed after a 4-week interval, while perioperative changes were evaluated intraoperatively. Changes in Hb were measured 24 h after surgery. Tissue retrieval percentage was calculated as the proportion of removed tissue relative to the preoperatively measured PV. Enucleation time was defined as the total time from the start of the procedure to the completion of enucleation of the two lateral lobes and the middle lobe.

To compare patients, we included demographic parameters such as age, body mass index (BMI), and total serum prostate-specific antigen (PSA, ng/mL). Intervention-related adverse events were classified according to the Clavien–Dindo classification (CDC) system (23).

### Statistical analysis

The first 100 consecutive pulsed ThuLEP cases performed by surgeons 1, 2, and 3 were prospectively evaluated. All calculations and statistical analyses were performed using IBM SPSS version 29.0 (IBM, Armonk, NY, USA). Graphing and visualization of the compared groups were carried out using GraphPad Prism version 10.2.3 (GraphPad Software Inc., San Diego, CA, USA). Preoperative, demographic, perioperative, and postoperative data are presented as median and interquartile range (IQR) for continuous variables and as percentages for categorial variables. The Shapiro–Wilk test was used to assess the normality of distribution. Fisher's exact test was used for univariate analyses, while the Mann–Whitney *U*-test was used for continuous variables. Data for efficiency analyses are presented as mean ± standard deviation (SD), along with group quartiles. When comparing patient cohorts, a two-way analysis of variance (ANOVA) was performed, except for adverse event comparisons, which were analyzed using the chi-square test and Fisher's exact test. All reported *p*-values were two-sided and deemed statistically significant if *p* < 0.05. To assess potential case-mix bias through volumes that may influence surgical efficacy, we compared median prostate volumes across surgeons within their specific subsets using two-way ANOVA.

The study aimed to collect real-world evidence on the learning curves of urology surgeons with different levels of experience following our previous implementation of a structured learning mentorship program (16). Therefore, cohort comparisons were performed descriptively, and additional inferential analyses were not intended for hypothesis testing.

## Results

### Demographic parameters and patient characteristics

Demographic parameters for each surgeon's patient cohort are summarized in Supplementary Table 1. In total, we included 300 patients, representing the first 100 consecutive cases for each surgeon. Analysis of demographic parameters showed no significant differences between the patients in the three groups in terms of age, prostate volume, median IPSS and QoL scores, BMI, Qmax, PVR, Hb, PSA density, American Association of Anesthesiologists (ASA) score, or the presence of indwelling urinary catheters.

### Surgical parameters

Supplementary Table 2 presents the analysis of perioperative surgical parameters for all three surgeons. Statistically significant differences were observed in total enucleation time, enucleation efficacy, and total laser energy. No differences were found among the three surgeons with respect to morcellation time, total resected tissue, and relative resected tissue.

**Figure 1:**
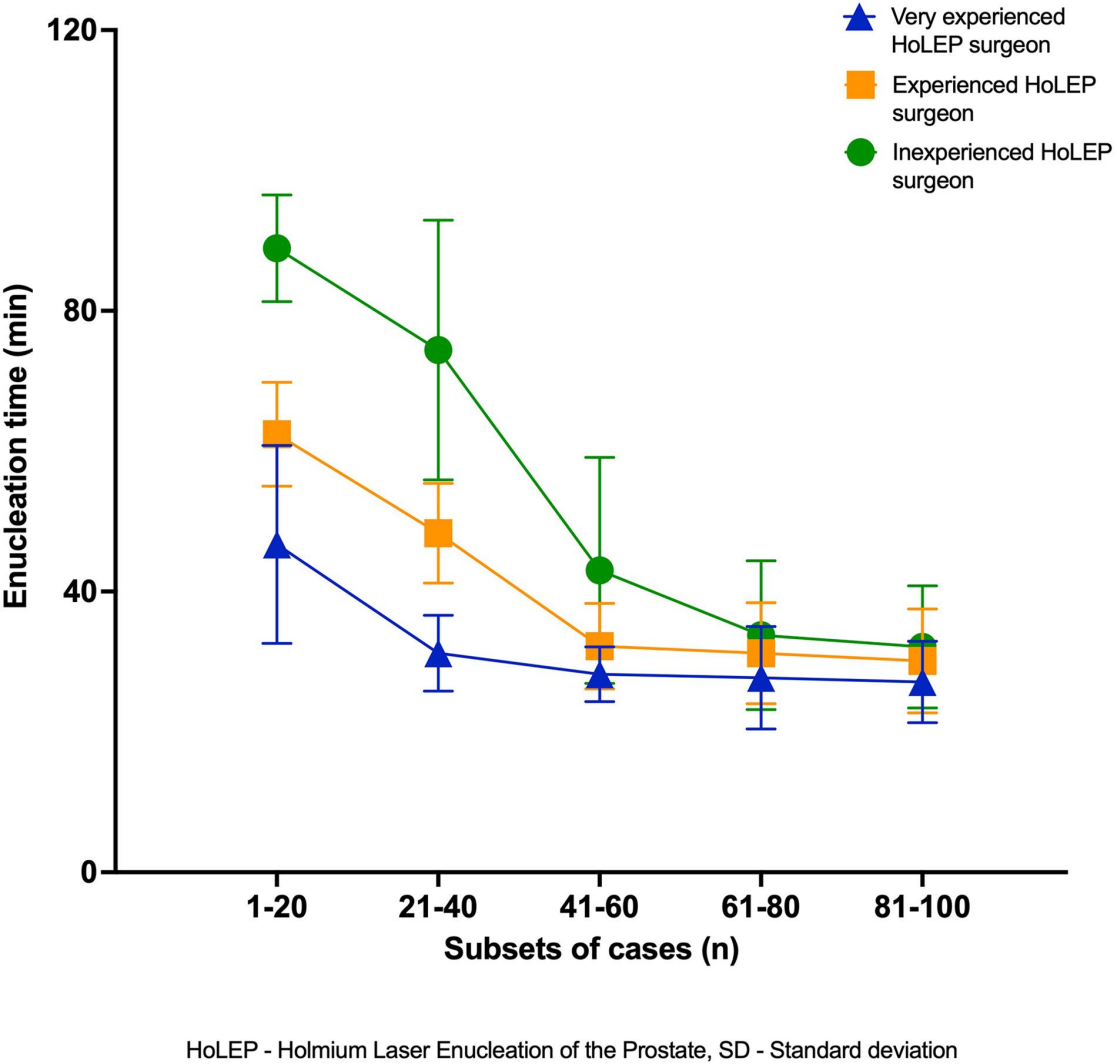
Enucleation time (min) over time with dispersion (SD).

**Figure 2:**
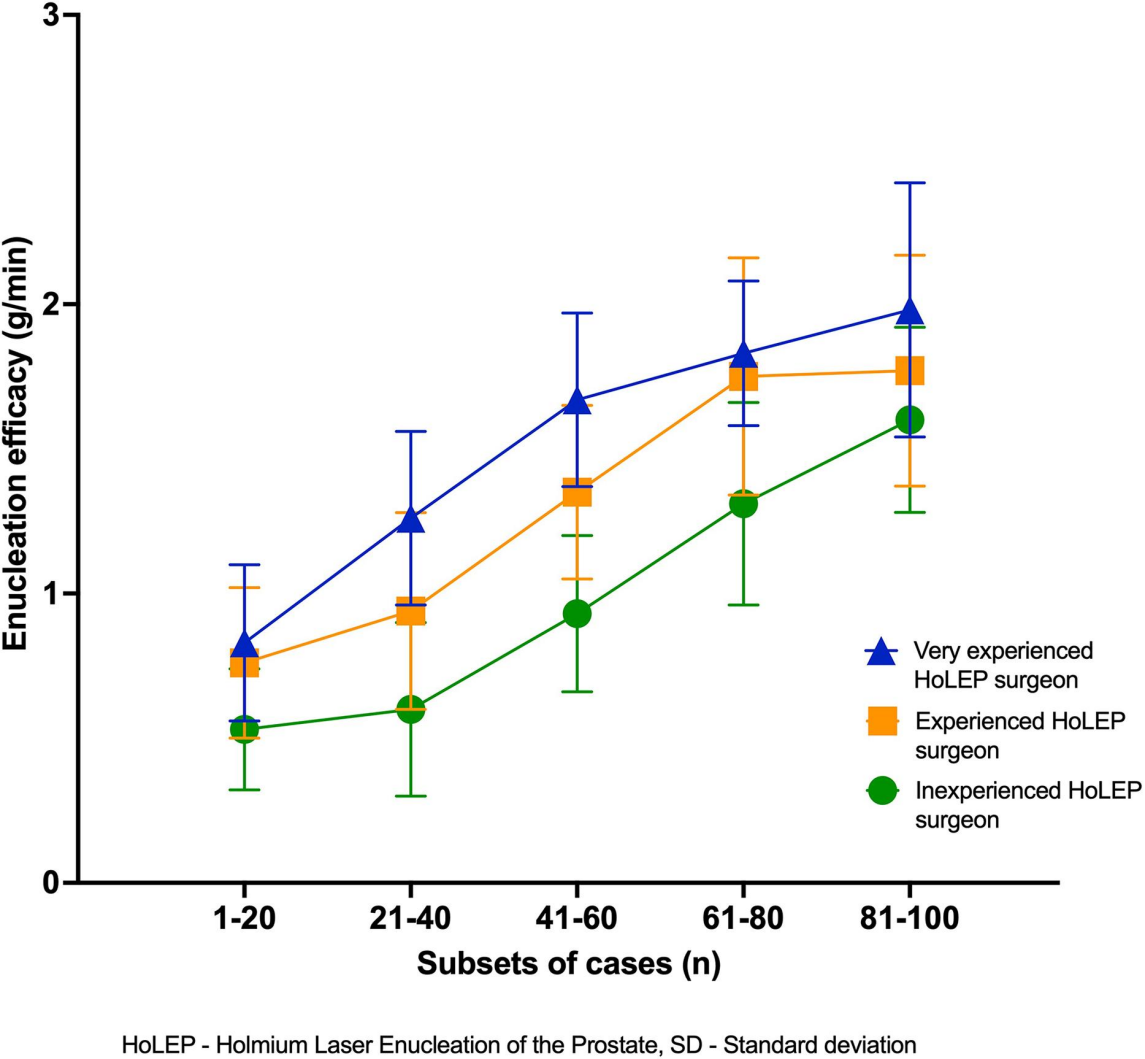
Enucleation efficacy (g/min) over time with dispersion (SD).

A more detailed analysis of the surgeons’ case-specific parameters is presented in Supplementary Tables 3A,B, showcasing the individual progression of each surgeon—adaptation to the new system for the experienced surgeons and learning for the TUR-P-experienced surgeon. These tables demonstrate an improvement along the learning curve, with statistically significant differences in perioperative parameters within the first 20 cases for the highly experienced surgeon, 40 cases for the experienced surgeon, and approximately 60 cases for the inexperienced surgeon to reach a plateau, with only minor improvements thereafter. Figures 1, 2 provide a graphical representation of the progression curves for the three surgeons, showing the points at which the surgeons reach a plateau in their learning curves, as demonstrated by the absence of significant difference between the case sets. We observed a plateau in enucleation time for the highly experienced surgeon after 40 cases, with no further significant decrease, while enucleation time improved by nearly 40%. Enucleation efficacy (Supplementary Table 2) reached a plateau after 60 cases, with efficacy more than doubling. The experienced and inexperienced surgeons reached their plateau in enucleation time after 60 and 80 cases, respectively, reducing their required time by approximately half (experienced surgeon) and nearly two-thirds (inexperienced surgeon) over the first 100 cases. Enucleation efficacy more than doubled during the observed time for the experienced surgeon, who reached a plateau after 80 cases, whereas it nearly tripled for the inexperienced surgeon, who did n't reach an observable plateau in efficacy within the first 100 cases.

**Figure 3:**
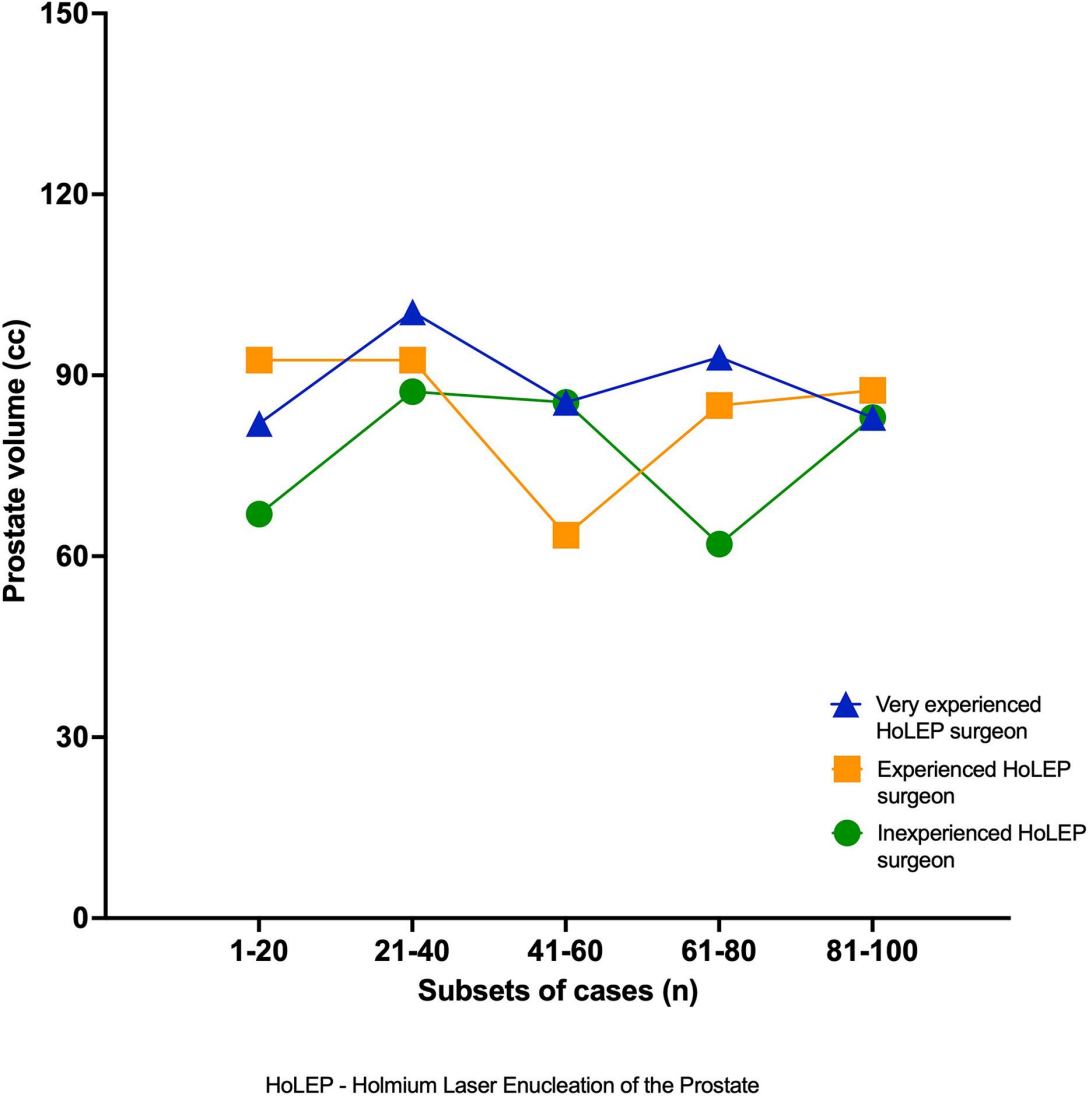
Median prostate volume (cm^3^) by case subset for each surgeon.

Supplementary Table 4 presents perioperative data on intraoperative laser energy requirements. Significant differences were observed for each surgeon when comparing the first and second halves of the 100 cases, as well as between different levels of experience. Comparison of median prostate volumes across all surgeons within their specific subsets showed no significant differences. The results are presented in Supplementary Table 6 and Figure 3.

### Perioperative and functional outcomes

Perioperative and functional clinical outcomes at 4 weeks postprocedure are presented in Supplementary Table 5. No significant differences were observed in IPSS, QoL, Qmax, and PVR among the three surgeons. Similarly, there were no differences in perioperative Hb decline. Postoperative complications (see Supplementary Table 7) did not differ significantly between the surgeons’ experience groups. Overall adverse events were observed in 19%, 18%, and 23% of cases (*p* = 0.65). Major complications (Clavien–Dindo ≥ III) were rare and comparable across groups (1%, 4%, and 4%; *p* = 0.36).

## Discussion

LUTS due to BPE represent a common medical condition in aging men, with incidence increasing due to demographic changes and a prevalence that increases almost linearly with age (24). To find an optimized treatment regimen for patients suffering from LUTS/BPO, current guidelines recommend a range of medical and surgical options, with the choice mostly depending on prostate size and patient characteristics such as comorbidities (5, 6, 9).

Among surgical treatment modalities, HoLEP has been proven to be size-independent, with superior efficacy compared with transurethral resection and a safety profile superior to that of open prostatectomy (OP) (10, 25–27). Thereby, it has earned its place in clinical guideline recommendations. However, with new systems entering the market, experienced surgeons will want to broaden their portfolios and incorporate these new technologies into teaching.

Since its introduction in 2010, Thulium laser enucleation of the prostate has become an established surgical method for the treatment of LUTS and has been assessed in terms of learning curves in structured training programs (6, 11, 12, 28). Pulsed ThuLEP enucleation is one of the newest developments in the family of laser enucleation systems. Unlike earlier Thulium laser systems, which used high-power continuous-wave systems, pulsed ThuLEP offers promising performance in tissue dissection through the pulsatile application of energy (17, 18). While preliminary studies have shown its efficacy and safety in smaller cohorts and among experienced surgeons, there is currently no study comparing learning curves across different levels of experience, nor any real-world study involving a patient cohort of comparable size (19).

No observable differences were observed between the patient cohorts operated on by the three surgeons. This represents a major strength of our study, as it delivers real-world data in a setting that reflects clinical reality. The cohorts, comprising the first 100 cases for all three surgeons, were not matched, as the aim was to achieve the best comparability in a realistic setting. Although there was a tendency for the two more experienced surgeons to operate on larger prostates, deemed unsuitable as learning cases, the cohorts still appeared similar enough to be comparable. To further rule out case-mix bias related to volumes that may have influenced surgical efficacy, we compared median prostate volumes across all surgeons within their specific subsets and found no significant differences that could have influenced the observed learning curves. In an earlier study by our group analyzing HoLEP learning curves, the mean prostate volume in the first 100 cases was approximately 90 cm^3^ (16). Perri et al. assessed the learning curve of a single surgeon with no prior laser enucleation experience but with TUR-P experience, reporting a mean prostate size of 89.7 cm^3^ for the first 100 cases (28). These findings are in accordance with the mean sizes operated on by our more experienced surgeons. The mean prostate size for the inexperienced surgeon was 74 cm^3^, which might suggest a better learning experience when operating on smaller prostates.

Enucleation times for the inexperienced surgeon across sets of 20 cases appear consistent with the results by Perri et al. Also, a resected tissue percentage of approximately two-thirds of the preoperative prostate volume aligns with previously published results.

Data on enucleation efficacy for surgeons experienced in one laser enucleation technique switching to another are limited. Himmler et al. reported a mean enucleation efficacy of 1.5 g/min for two ThuLEP-experienced surgeons switching to HoLEP in their first 50 consecutive cases, starting at 1 g/min in the first set of 10 cases, with no significant learning curve observed (21). While the mean enucleation efficacy reported in that study is comparable to our data, we found a significant learning curve, likely attributable to our larger cohort size.

The enucleation efficacy learning curve for the inexperienced surgeon (Supplementary Table 3) is in accordance with the data presented by Perri et al., with enucleation efficacy improving approximately by half with each set of 20 cases until reaching a plateau (28). However, our data for the inexperienced surgeon unfortunately fails to reach a plateau, hinting at further improvement beyond the observed timeframe.

In concordance with data from earlier studies evaluating learning curves in similar laser enucleation systems, our study shows that pulsed ThuLEP can be learned within an adequate timeframe when performed within a structured training program at a center with appropriate case numbers (15, 16, 21, 28). Furthermore, it is safe for patients regardless of the level of prior experience of their treating surgeon. Our data indicate that greater prior experience in laser enucleation is associated with a shorter switching period when adopting a new system. The highly experienced surgeon needed only 20 cases to reach a plateau, while our experienced surgeon reached this plateau after 40 cases. In contrast, a surgeon experienced in endourologic procedures but without prior experience in laser enucleation reaches a plateau after approximately 60 cases when trained within a structured program that accelerates learning (16). Experience with other laser enucleation methods also results in a higher plateau achieved by more experienced surgeons compared with their less experienced colleagues. In accordance with prior studies focusing on patient safety, we found no significant differences in surgical or functional outcomes between surgeons with different levels of experience, including quality of life, blood loss, and functional improvement (29). These findings highlight the safety of the technique and demonstrate that pulsed Thulium enucleation can be taught safely and efficiently within a structured training program. A limitation in the assessment of functional outcomes is the relatively short follow-up period of only 4 weeks. Irritative symptoms or incontinence, which have been reported by Wu et al. to develop at a later point, might not have been adequately captured, as our study focused on an early learning-curve analysis (30). Conversely, existing evidence from Elshal et al. showed no relevant difference between short-term (30-day) and long-term (1-year) functional outcomes after transurethral laser surgery for BPH (31). Therefore, while no changes in functional outcomes are expected, definite long-term functional benefits cannot be drawn from our data and would require a longer follow-up. Nevertheless, this fact makes the system an attractive alternative for surgeons or centers seeking to expand their portfolio of treatment options that can be offered to patients.

Our data show a significant decrease in total laser energy required over the course of the first 100 consecutive cases for all surgeons. Although the ecologic and economic benefits of lower energy consumption are obvious, the clinical relevance of the perioperative laser energy required remains a topic of discussion. While data from large patient cohorts suggest that laser energy have no significant influence on functional or clinical outcomes (32), smaller cohorts indicate that higher perioperative laser energy may negatively impact postoperative erectile function (33). Unfortunately, our study design did not assess postoperative erectile function; therefore, it can neither confirm nor refute these assumptions.

Our findings are in accordance with the current scientific body of knowledge, indicating that pulsed ThuLEP is at least equivalent to the gold standard, HoLEP, among other alternatives (6, 12, 19, 21).

Overall, although exploratory, our data demonstrate that pulsed Thulium laser enucleation is safe and feasible to be learned by surgeons experienced in HoLEP without the need for extensive training. Moreover, it can be safely and rationally incorporated into training programs for surgeons experienced in endourological procedures, despite a steep learning curve, without increasing complication rates or compromising functional outcomes for patients. Thus, our study contributes valuable data to the existing literature, showing that pulsed ThuLEP can be a safe and feasible addition to a surgeon's portfolio.

## Conclusion

In the present study, we demonstrated that transurethral pulsed ThuLEP laser enucleation of the prostate is an effective and safe treatment option for LUTS secondary to BPE. Favorable peri- and postoperative outcomes, along with a good learning curve for HoLEP-experienced surgeons, make it a valuable addition to the range of treatment options for LUTS patients. Future studies involving larger patient cohorts are needed to assess long-term outcomes and compare this technique with other established methods.

## Data Availability

The raw data supporting the conclusions of this article will be made available by the authors upon reasonable request, without undue reservation.
