# Supplementary material for: Switching lasers: assessing the learning curves of surgeons with different levels of surgical experience when switching from HoLEP to pulsed Thulium YAG lasers for ThuLEP
Source: Front Surg. 2026 Apr 13;13:1799916. doi: 10.3389/fsurg.2026.1799916 (PMC13111452; doi:10.3389/fsurg.2026.1799916)
Supplement: Supplementary file 1 [file Table1.docx]

| Table 1 – patient characteristics | | | | | |
| --- | --- | --- | --- | --- | --- |
| Variables | Overall | Very experienced Holep surgeon | Holep Experienced surgeon | Inexperienced Holep surgeon | p-value |
| Age (years)  Median  IQR | 71.0  (63.0 – 76.0) | 67.0  (59.5 – 75.0) | 70.0  (63.0 – 79.0) | 65.5  (63.0 – 69.8) | 0.435 |
| Prostate volume (cc)  Median  IQR | 94.5  (68.0 – 123.0) | 90.0  (60.0 - 120.0) | 82.5  (60.5 - 122.5) | 74.0  (60.0 – 91.3) | 0.118 |
| IPSS  Median  IQR | 21  (16 - 25) | 22  (16 - 27.5) | 21  (14 - 23.75) | 20.5  (14.4 – 24.25) | .383 |
| QoL  Median  IQR | 5  (3-5) | 4  (3 - 4) | 4  (3 - 4.75) | 4  (3 – 4.25) | .982 |
| BMI (kg/m²)  Median  IQR | 26.0  (24.1 – 29.0) | 26.7  (23.9 - 29.1) | 25.1  (23.1 – 28.4) | 25.82  (23.9 – 28.3) | .596 |
| Qmax (ml/s)  Median  IQR | 7.6  (5.9 – 11.0) | 9.0  (6.7 - 13.8) | 8.1  (5.9 – 11.0) | 7.6  (6.4 – 11.0) | .364 |
| PVR (ml)  Median  IQR | 80  (50 – 127) | 90  (45 – 135) | 80  (50 – 120) | 80  (50 – 150) | .528 |
| Hb (g/dl)  Median  IQR | 14.8  (13.8 – 15.6) | 14.9  (14.3 - 15.4) | 14.8  (13.9 -15.6) | 15.4  (14.4 – 15.8) | .322 |
| Total PSA (ng/ml)  Median  IQR | 5.39  (2.66 – 9.64) | 5.07  (2.14 - 10.34) | 4.34  (2.56 – 7.37) | 4.38  (2.63 – 6.22) | .039 |
| PSA density (ng/ml/cc) Median  IQR | 0.0583  (0.036 – 0.090) | 0.062  (0.353 - 0.091) | 0.042  (0.038 – 0.086) | 0.056  (0.040 – 0.085) | .180 |
| ASA I (% (n))  ASA II (% (n))  ASA III (% (n))  ASA IV (% (n)) | 9.7 % (29)  59.2 % (177)  29.7 % (89)  1.3 % (4) | 7.1 % (7)  60.6 % (60)  31.3 % (31)  1.0 % (1) | 11.0 % (11)  60.0 % (60)  27.0 % (27)  2.0 % (2) | 11.0 % (11)  57.0 % (57)  31.0 % (31)  1.0 % (1) | .756 |
| IDC (%)  n | 35.4 %  105 | 39.4 %  39 | 37.4 %  37 | 29.3 %  29 | .290 |
| ASA - American society of Anesthesiologists, BMI - body mass index, Hb – hemoglobin, HoLEP – Holmium Laser Enucleation of the Prostate, IDC - indwelling urinary catheter, IPSS - international prostate symptom score, IQR - interquartile range, PSA - prostate specific antigen, PVR - postvoid residual urine volume, Qmax - peak urinary flow rate, QoL - quality of life; | | | | | |
