# Supplementary material for: Switching lasers: assessing the learning curves of surgeons with different levels of surgical experience when switching from HoLEP to pulsed Thulium YAG lasers for ThuLEP
Source: Front Surg. 2026 Apr 13;13:1799916. doi: 10.3389/fsurg.2026.1799916 (PMC13111452; doi:10.3389/fsurg.2026.1799916)
Supplement: Supplementary file 2 [file Table2.docx]

| Table 2 – Surgical parameters | | | | |
| --- | --- | --- | --- | --- |
| Variables | Very experienced Holep surgeon | Holep Experienced surgeon | Inexperienced Holep surgeon | p-value |
| Enucleation time (min)  Median  IQR | 29.5  (26.3 – 36.0) | 36.6  (28.5 – 50.9) | 45.7  (32.0 – 80.4) | <0.001* |
| Enucleation efficacy (g/min)  Median  IQR | 1.57  (1.14 – 1.80) | 1.36  (0.86 – 1.64) | 0.93  (0.53 – 1.42) | <0.001* |
| Morcellation time (min)  Median  IQR | 6.0 (n = 7)  (5.0 – 8.0) | 5.5 (n = 6)  (2.75 – 11.25) | 7.0 (n = 4)  (3.0 – 11.75) | 0.830 |
| Resected tissue (g)  Median  IQR | 60.5  (42.0 – 79.5) | 63.5  (36.9 – 86.0) | 50.0  (35.0 – 75.0) | 0.122 |
| Resected tissue (%)  Median  IQR | 62.6  (50.1 – 75.0) | 59.7  (50.0 – 73.9) | 58.3  (48.7 – 74.6) | 0.620 |
| Laser energy (kJ)  Median  IQR | 63.9  (53.6 – 75.9) | 76.0  (65.8 – 88.4) | 98.8  (86.1 – 112.3) | <0.001* |
| HoLEP – Holmium Laser Enucleation of the Prostate, IQR - interquartile range; | | | | |
