# Supplementary material for: Switching lasers: assessing the learning curves of surgeons with different levels of surgical experience when switching from HoLEP to pulsed Thulium YAG lasers for ThuLEP
Source: Front Surg. 2026 Apr 13;13:1799916. doi: 10.3389/fsurg.2026.1799916 (PMC13111452; doi:10.3389/fsurg.2026.1799916)
Supplement: Supplementary file 3 [file Table3.docx]

| Table 3A - Surgical outcomes. enucleation time | | | |
| --- | --- | --- | --- |
| Enucleation time (min) | Very experienced Holep surgeon | Holep Experienced surgeon | Inexperienced Holep surgeon |
| Cases 1-20  (SD) | 46.7  (14.1) | 62.4  (7.4) | 88.9  (7.6) |
| Cases 21-40  (SD)  p-Value** | 31.2  (5.4)  <0.001* | 48.3  (7.1)  <0.001* | 74.4  (18.5)  <0.001* |
| Cases 41-60  (SD)  p-Value** | 28.2  (3.9)  0.052 | 32.2  (6.1)  <0.001* | 43.0  (16.1)  <0.001* |
| Cases 61-80  (SD)  p-Value** | 27.7  (7.3)  0.678 | 31.2  (7.2)  0.620 | 33.8  (10.6)  0.038* |
| Cases 81-100  (SD)  p-Value** | 27.1  (5.8)  0.738 | 30.1  (7.4)  0.445 | 32.1  (8.7)  0.414 |
| **p-Values in comparison to prior 20 consecutive cases  HoLEP – Holmium Laser Enucleation of the Prostate, SD – Standard Deviation; | | | |

| Table 3B - Surgical outcomes. enucleation efficacy | | | |
| --- | --- | --- | --- |
| Enucleation efficacy (g/min) | Very experienced Holep surgeon | Holep Experienced surgeon | Inexperienced Holep surgeon |
| Cases 1-20  (SD) | 0.83  (0.27) | 0.76  (0.26) | 0.53  (0.21) |
| Cases 21-40  (SD)  p-Value** | 1.26  (0.30)  <0.001* | 0.94  (0.34)  0.068 | 0.60  (0.30)  0.478 |
| Cases 41-60  (SD)  p-Value** | 1.67  (0.30)  <0.001* | 1.35  (0.30)  <0.001* | 0.93  (0.27)  <0.001* |
| Cases 61-80  (SD)  p-Value** | 1.83  (0.25)  0.096 | 1.75  (0.41)  <0.001 | 1.31  (0.35)  <0.001* |
| Cases 81-100  (SD)  p-Value** | 1.98  (0.44)  0.369 | 1.77  (0.40)  0.904 | 1.60  (0.32)  0.012* |
| **p-Values in comparison to prior 20 consecutive cases  HoLEP – Holmium Laser Enucleation of the Prostate, SD – Standard Deviation; | | | |
