# Supplementary material for: Switching lasers: assessing the learning curves of surgeons with different levels of surgical experience when switching from HoLEP to pulsed Thulium YAG lasers for ThuLEP
Source: Front Surg. 2026 Apr 13;13:1799916. doi: 10.3389/fsurg.2026.1799916 (PMC13111452; doi:10.3389/fsurg.2026.1799916)
Supplement: Supplementary file 4 [file Table4.docx]

| Table 4 – Laser energy comparison between surgeons | | | |
| --- | --- | --- | --- |
| Laser energy (kJ) | First 50 cases | Last 50 cases | p-Value between first and last cases |
| Surgeon 1  Mean  (SD) | 70.6  (20.0) | 62.3  (17.8) | 0.031* |
| p-Value comparison  Surgeon 1 – Surgeon 2 | First 50 cases  0.002* | Last 50 cases  0.004* |  |
| Surgeon 2  Mean  (SD) | 88.2  (26.7) | 72.6  (16.0) | 0.002* |
| p-Value comparison  Surgeon 2 -Surgeon 3 | First 50 cases  <0.001* | Last 50 cases  <0.001* |  |
| Surgeon 3  Mean  (SD) | 108.0  (20.9) | 93.3  (15.6) | <0.001* |
| p-Value comparison  Surgeon 3 – Surgeon 1 | First 50 cases  <0.001* | Last 50 cases  <0.001* |  |
| Table 4 showcasing the differences in laser energy needed, surgeon 1 being the very experienced, surgeon 2 the Holep-experienced and surgeon 3 the inexperienced surgeon  HoLEP – Holmium Laser Enucleation of the Prostate, SD – Standard deviation; | | | |
