# Supplementary material for: Switching lasers: assessing the learning curves of surgeons with different levels of surgical experience when switching from HoLEP to pulsed Thulium YAG lasers for ThuLEP
Source: Front Surg. 2026 Apr 13;13:1799916. doi: 10.3389/fsurg.2026.1799916 (PMC13111452; doi:10.3389/fsurg.2026.1799916)
Supplement: Supplementary file 5 [file Table5.docx]

| Table 5 – Functional Outcomes | | | | |
| --- | --- | --- | --- | --- |
| Variables | Very experienced Holep surgeon | Holep-Experienced surgeon | Inexperienced Holep surgeon | p-value |
| Δ IPSS  Median  IQR | 11  (5 – 16) | 10  (6 – 15) | 9  (5 – 13) | .701 |
| Δ QoL  Median  IQR | 2  (1 – 2) | 2  (1 – 3) | 2  (1 – 3) | .608 |
| Δ Qmax (ml/s)  Median  IQR | 5.1  (3.0 – 12.3) | 5.6  (3.5 – 9.8) | 4.8  (3.5 – 9.3) | .790 |
| Δ PVR (ml)  Median  IQR | 70.0  (27.5 – 122.5) | 60.0  (37.5 – 105.0) | 75.0  (38.8 – 132.5) | .543 |
| Δ Hb (g/dl)  Median  IQR | 1.1  (0.7 – 2.0) | 1.4  (0.6 – 2.1) | 1.4  (0.5 – 2.2) | .652 |
| Hb – hemoglobin, HoLEP – Holmium Laser Enucleation of the Prostate, IPSS - international prostate symptom score, IQR - interquartile range, PVR - postvoid residual urine volume, Qmax - peak urinary flow rate, QoL - quality of life; | | | | |
