# Supplementary material for: Switching lasers: assessing the learning curves of surgeons with different levels of surgical experience when switching from HoLEP to pulsed Thulium YAG lasers for ThuLEP
Source: Front Surg. 2026 Apr 13;13:1799916. doi: 10.3389/fsurg.2026.1799916 (PMC13111452; doi:10.3389/fsurg.2026.1799916)
Supplement: Supplementary file 6 [file Table6.docx]

| Table 6 – Prostate Volume per subset | | | |  |
| --- | --- | --- | --- | --- |
| Median Prostate volume (cc) | Very experienced Holep surgeon | Holep- Experienced surgeon | Inexperienced Holep surgeon | p-value |
| Cases 1-20  (SD) | 82.0  (39.1) | 92.5  (46.9) | 67.0  (42.7) | 0.394 |
| Cases 21-40  (SD) | 100.5  (39.2) | 92.5  (39.2) | 87.3  (77.6) | 0.659 |
| Cases 41-60  (SD) | 85.5  (61.6) | 63.5  (41.4) | 85.5  (57.6) | 0.724 |
| Cases 61-80  (SD) | 93.0  (33.6) | 85.0  (59.6) | 62.0  (46.9) | 0.248 |
| Cases 81-100  (SD) | 83.0  (34.7) | 87.5  (52.8) | 83.0  (42.1) | 0.757 |
| HoLEP – Holmium Laser Enucleation of the Prostate, SD – Standard deviation | | | | |
