# Supplementary material for: Switching lasers: assessing the learning curves of surgeons with different levels of surgical experience when switching from HoLEP to pulsed Thulium YAG lasers for ThuLEP
Source: Front Surg. 2026 Apr 13;13:1799916. doi: 10.3389/fsurg.2026.1799916 (PMC13111452; doi:10.3389/fsurg.2026.1799916)
Supplement: Supplementary file 7 [file Table7.docx]

| Table 7 – Complications as per Clavien-Dindo-Classification (CDC) | | | | |  |
| --- | --- | --- | --- | --- | --- |
| CDC Grade | Very experienced Holep surgeon  (n = 100) | Holep-Experienced surgeon  (n = 100) | Inexperienced  Holep surgeon  (n = 100) | p-value | |
| Overall AEs  n (%) | 19 (19) | 18 (18) | 23 (23) | 0.65 | |
| Grade I  Urinary retention  Macrohematuria  Bladder injury  n (%) | 4  13  1  18 (18) | 5  7  1  13 (13) | 4  10  2  (16) | 0.62 | |
| Grade II  Blood transfusion  n (%) | 0  0 (0) | 1  1 (1) | 2  2 (2) | 0.36 | |
| Grade IIIa  Clot retention  Grade IIIb  Surgical coagulation  n (%) | 0  1  1 (1) | 2  2  4 (4) | 3  1  4 (4) | 0.36 | |
| Grade IV  Circulatory failure  n (%) | 0  0 (0) | 0  0 (0) | 1  1 (1) | 0.37 | |
| Grade V  n (%) | 0 (0) | 0 (0) | 0 (0) | n.a | |
| CDC – Clavien-Dindo-Classification, HoLEP – Holmium Laser Enucleation of the Prostate; | | | | | |
